# Executive functioning challenges of adolescents born extremely and very preterm

**DOI:** 10.3389/fpsyg.2024.1487908

**Published:** 2024-12-11

**Authors:** Samantha J. Lee, Lianne J. Woodward, Stephanie Moor, Nicola C. Austin

**Affiliations:** ^1^School of Health Sciences and Canterbury Child Development Research Group, University of Canterbury, Christchurch, New Zealand; ^2^Older Person’s Mental Health, Burwood Hospital, Christchurch, New Zealand; ^3^Department of Paediatrics, University of Otago Christchurch, Christchurch, New Zealand; ^4^Neonatal Unit, Christchurch Women’s Hospital, Christchurch, New Zealand

**Keywords:** very preterm birth, neonatal risk, medical complexity, executive function, neurodevelopmental outcome, adolescence

## Abstract

**Background:**

Children born very preterm (VPT; <32 weeks) are at increased risk of executive functioning (EF) difficulties. But less is known about the nature and extent of these executive difficulties during late adolescence, particularly across multiple EF domains and in response to varying degrees of executive demand.

**Methods:**

Using data from a prospective longitudinal study, this paper describes the EF profiles of 92 VPT and 68 full-term (FT) adolescents at age 17 years. Relations between gestational age (GA) and later EF performance, in addition to neonatal predictors, were examined.

**Results:**

VPT-born adolescents performed less well than FT adolescents across the domains of working memory, planning, and cognitive flexibility, with the largest differences observed for those born <28 weeks GA (effect sizes −0.6 to −1.0 SD), and when task demands were high. The effects of GA on EF outcome were fully mediated by neonatal medical complexity (*b* = 0.169, *t* = −1.73) and term equivalent white matter abnormalities (*b* = 0.107, *t* = −3.33).

**Conclusion:**

Findings support the need for long-term cognitive support for individuals born very preterm, particularly those exposed to high levels of medical and neurological risk, with these factors largely explaining associations between GA and EF outcome.

## 1 Introduction

Advances in neonatal care have significantly improved survival for children born very preterm (VPT; <32 weeks), with the most dramatic gains seen amongst those born at the lower limits of viability (Higgins et al., 2024; Morgan et al., 2022). But despite these gains, rates of adverse neurodevelopmental outcomes for VPT infants remain stable (Cheong et al., 2017). These include cerebral palsy, developmental co-ordination disorder, blindness, deafness, attention deficit-hyperactivity disorder, autism spectrum disorder, and educational underachievement (Broring et al., 2018; Cheong et al., 2017; Doyle et al., 2021; Jois, 2019; Pierrat et al., 2021).

By far, the most common adverse neurodevelopmental outcome is cognitive impairment. Meta-analyses indicate that children and adolescents between the ages of 4 and 20 years consistently obtain IQ scores that are more than 0.8 SDs below their full-term (FT) peers (Brydges et al., 2018; Twilhaar et al., 2018), with between 40 and 50% experiencing mild to severe cognitive impairment (Doyle et al., 2021; Erdei et al., 2020; Pierrat et al., 2021). Executive functioning (EF) difficulties are also common during early and middle childhood. Executive functions consist of a range of interrelated top-down cognitive skills that enable an individual to achieve a desired goal. Key domains include inhibitory control, working memory, planning, and cognitive flexibility (Nigg, 2017). Findings suggest that between the ages of 4 and 14 years, mean score differences between VPT and FT-born children range from 0.39 to 0.52 SDs across different EF-related tasks (van Houdt et al., 2019).

Less is known about the EF performance of VPT children during late adolescence. Yet, adolescence is a critical period of brain development marked by dramatic neurological changes alongside rapid increases in learning, independence, and social change (Blakemore and Choudhury, 2006; Steinberg, 2005). It is also a time when executive difficulties may have pervasive impacts on daily functioning and life course opportunities (Larsen and Luna, 2018). Data generally suggest that during this developmental stage, individuals born VPT tend to perform less well than FT controls across multiple EF measures, but with varying magnitudes of performance detriment (Burnett et al., 2015; Lundequist et al., 2015; Luu et al., 2011; Stalnacke et al., 2019; Taylor et al., 2004). However, there is considerable variability across existing studies on the conceptualization and measurement of EF, with some studies utilizing a single measure to assess one EF skill and comparatively few employing multiple measures across different EF domains. Further, the effects of varying degrees of task difficulty on EF performance at this age are unclear. Adolescents born VPT are more likely to experience educational and social challenges (Twilhaar et al., 2019; Wolke et al., 2013), which may reflect difficulty coping with the increased EF demand. Thus, a detailed assessment of how changing task demands may impact neurocognitive performance could help further our understanding of the nature of the difficulties some individuals may experience, and potentially the conditions under which risks may be greatest and the various ways these problems may manifest.

In the current study, we used the unity/diversity (Miyake and Friedman, 2012) and executive control system frameworks (Anderson, 2002) to inform our operationalization and measurement selection to characterize the EF profile of 17-year-old adolescents who were born VPT. Three core executive domains shown to have different developmental trajectories from early childhood to adolescence were assessed, spanning working memory, planning, and cognitive flexibility (Diamond, 2013). We examine these domains at age 17 since this age marks an important transition point between adolescence and young adulthood that is associated with increasing autonomy. For those with developmental challenges, it can be a particularly complex stage given the range and level of cognitive skills involved in making a successful transition to adult roles and responsibilities (Leebens and Williamson, 2017). Finally, EF measures were also selected to allow an examination of the extent to which EF performance varied with increasing task difficulty or cognitive demand and to minimize possible floor or ceiling effects.

A further issue of interest is the early identification of those VPT born infants that may be at greatest risk of EF challenges, since timely intervention is critical to mitigate long-term impacts. The varying degrees of executive dysfunction observed across previous cohorts suggests that later EF challenges were more pronounced for adolescents born extremely preterm (EPT; <28 weeks) (Burnett et al., 2015; Farooqi et al., 2016; Lundequist et al., 2015; Taylor et al., 2004). However, the extent to which these elevated risks are explained by infant neonatal medical and postnatal factors has been less well studied. Most studies to date have separately examined a range of individual medical factors, with findings inconsistent across studies and results varying depending on the outcome measures used. Medical factors linked with lower EF performance include ventilation requirement, chronic lung disease, inflammation/infection, and abnormal EEG or cranial ultrasound results (Leviton et al., 2018; Luu et al., 2011; Saavalainen et al., 2007; Taylor et al., 2004). Yet, in reality, many neonatal medical risk factors are highly comorbid. They are also likely to have potentially cumulative impacts on child outcomes, with later risks increasing with higher levels of neonatal medical risk (Barnett et al., 2018). Thus, a cumulative medical risk index may be a better approach in terms of studying the impacts of neonatal medical adversity when identifying individuals at later EF risk.

Furthermore, few adolescent EF studies have included measures of early neurological risk. A strong association between the extent of white matter abnormalities and preschool and school-age neurocognitive outcomes was found in the current cohort (Woodward et al., 2012; Woodward et al., 2011), and other VPT samples (Anderson et al., 2017; Iwata et al., 2012). The presence of neonatal white matter abnormalities is, therefore, considered as another potentially useful predictor of EF problems that persist into adolescence.

**Figure 1:**
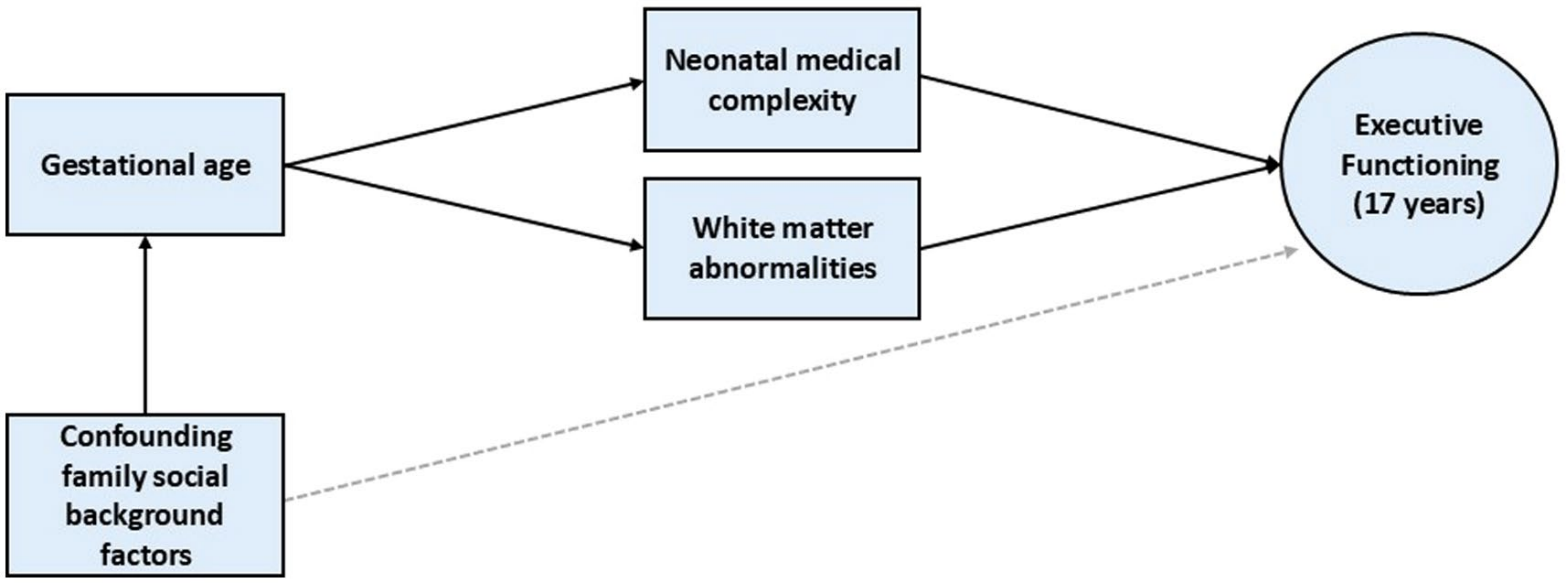
Hypothesized pathways from gestational age to executive functioning outcome at age 17 years.

Taken together, the specific study aims were as follows:

1. To compare the EF performance of EPT (23–27 weeks), VPT (28–32 weeks) and FT comparison adolescents at age 17 years across three domains including working memory, planning, and cognitive flexibility. Also of interest was the extent to which increasing cognitive demand might impact EF performance for each group, and whether between-group differences might also be explained by family social background factors correlated with very preterm birth.
2. To examine the extent to which neonatal medical complexity and the presence of white matter abnormalities by term equivalent might mediate associations between gestational age (GA) and EF performance at age 17 years. Hypothesized pathways between these variables of interest are shown in Figure 1.

## 2 Materials and methods

### 2.1 Participants

Participants included two groups of adolescents drawn from a prospective longitudinal study in Christchurch, New Zealand. The first group was a regional cohort of 110 children born ≤33 weeks gestation and/or ≤ 1,500 g who were consecutively admitted to a level III neonatal intensive care unit at Christchurch Women’s Hospital from November 1998 to December 2000 (92% recruitment). Children with congenital abnormalities and non-English speaking parents were excluded. Follow-up assessments were conducted at corrected age 2, 4, 6, 9, and 12 years. Excluding deaths (*n* = 3), 86% (*N* = 92) of these participants completed the 17-year follow-up assessment. Reasons for non-participation included declined (*n* = 12), and ineligible (did not meet original study criteria, *n* = 3). There was no significant difference in the mean GA or family socioeconomic status (SES) between participants and non-participants. For the current analysis, the VPT group was further stratified into those born EPT (23–27 weeks) and VPT (28–32 weeks).

The second group comprised 113 full-term born (38–41 weeks gestation) adolescents who were recruited at age 2 years (62% recruitment), and then assessed alongside the VPT group. These full-term control group participants were identified from hospital birth records by selecting a same-sex child born two births before or two births after the delivery of each VPT participant. Exclusion criteria were congenital abnormalities, foetal alcohol effects, birth complications such as growth restriction, and non-English speaking parents. A comparison of the socioeconomic profile of this group with regional census data showed that it was highly representative of the recruitment region. A total of 68 full-term controls were seen at the 17-year follow-up (60% retention). Reasons for attrition included declined participation (*n* = 27), withdrawal (atypical development precluded further participation, *n* = 4), ineligible (did not meet original study criteria, *n* = 1), relocation, with funding restrictions preventing invitation to participate (*n* = 15), and severe illness (*n* = 1). There was no significant difference in family SES between participants and non-participants.

### 2.2 17-year procedure

All procedures and measures were approved by the Southern Health and Disability Ethics Committee (ref: 14/STH/208), and all adolescent participants and their parents/caregivers provided written informed consent. At age 17, participants underwent neuropsychological testing as part of a larger multidisciplinary one-day follow-up assessment, including an extensive battery of EF measures. One clinical psychologist blinded to the participant’s group status administered the test battery in the same predetermined order.

### 2.3 17-year EF measures

Four EF tasks from the Cambridge Neuropsychological Test Automated Battery (CANTAB) (Cambridge Cognition, 2013) were administered on a 10.1-inch touchscreen tablet. Working memory was assessed with the Spatial Span (SSP) and Spatial Working Memory (SWM) tasks, planning abilities were assessed with the Stockings of Cambridge (SOC) task, and cognitive flexibility was assessed with the Intra-Extra Dimensional Set Shift (IED) task. The Comprehensive Trail-Making Test (CTMT) (Reynolds, 2002) was administered as an additional measure of cognitive flexibility. Further description of each of these tasks and the outcome measures used in the analysis are provided in Table 1.

**Table 1:** Description of executive function measures and key variables.

Test name	Test description	Key dependent variables
Working memory		
CANTAB Spatial span (SSP)	Adapted Corsi Blocks task. Participant is required to recall a sequence of boxes that change color one by one. Trials begin at 2-box sequences and continue up to 9-box sequences. In the backward variant, boxes must be selected in the reverse order that they were displayed.	(1) Span length: the max. Sequence recalled (higher score = better spatial span)
CANTAB Spatial working memory (SWM)	Adapted self-ordered search task. Participants search a number of boxes to uncover a hidden “token.” Trial continues until all tokens are found. Four trials each consisting of 4-, 6-, and 8-boxes to search under are assessed, for a total of 12 trials.	(1) Total revisit errors: times a box already found containing a token was selected (lower scores = fewer errors)
Planning		
CANTAB Stockings of Cambridge (SOC)	Adapted Tower of London task. Participants must move balls to recreate a target display in a specified number of moves. There are two trials each for the 2- and 3-move levels, and four trials each for the 4- and 5-move levels. Trials are terminated if unsolved after more than double the minimum moves have been executed.	(1) % of problems solved in the specified number of moves or “perfect solutions” (higher = more problems solved) (2) planning time before moving first ball (longer = greater planning time)
Cognitive flexibility		
CANTAB Intra-extra dimensional set shift (IED)	Adapted Wisconsin Card Sorting task. A forced-choice discrimination test of rule acquisition and reversal. Participants select an image and, through trial and error, discover the rule that determines which image is correct. Following six consecutive correct choices, the rule then changes without notice. Rule shifts in the initial stages are intra-dimensional stimulus shifts and in the later stages are extra-dimensional shifts.	(1) Total number of errors made, adjusted for total stages completed (2) Intra-dimensional shift errors (3) Extra-dimensional shift errors (lower scores = fewer errors made)
Comprehensive trail-making test (CTMT)	A pen and paper test comprised of five visual search and sequencing trails. Participants must connect a series of stimuli in a predetermined order as quickly as possible. The trails become increasingly difficult by including distractor stimuli, and incorporating different stimuli into the sequence.	(1) Total time taken to complete the sequence (faster = better) (2) Composite Index T score (higher score = better overall performance)

### 2.4 Infant clinical characteristics

Infant clinical data, including GA, sex and medical information were gathered from hospital records. A continuous neonatal medical complexity index was also created by identifying six major infant medical exposures that were experienced during the NICU stay. These included: level of respiratory support; time to full enteral feeds; severe retinopathy of prematurity; neonatal sepsis including necrotizing enterocolitis (NEC): intraventricular haemorrhage/periventricular leukomalacia (IVH/PVL); and any major surgery. Scores from 0 to 2 were assigned to each medical exposure based on severity, with scores summed to provide an overall neonatal medical complexity score (see Supplementary Table S1 for further details).

### 2.5 Neonatal white matter abnormality

All EPT and VPT infants underwent structural MR imaging at term equivalent age (39–41 weeks gestation), using a 1.5-tesla General Electric Signa System. MRI at term equivalent was used given its predictive accuracy in identifying high-risk children who may benefit from surveillance and targeted early intervention. Each infant’s scan was scored by a blinded pediatric neuroradiologist and independently reviewed by a pediatric neurologist (95% inter-rater agreement) on the following scales: the presence and severity of periventricular white matter volume loss, white matter signal abnormality, the presence of cystic abnormalities, ventricular dilation, and thinning of the corpus callosum and reduced myelination (See Woodward et al. (2006) for further details). Based on their total white matter abnormality scores, children were classified as follows: (1) no abnormalities (score of 5 to 6); (2) mild abnormalities (score of 7 to 9); (3) moderate abnormalities (score of 10 to 12); or (4) severe abnormalities (score > 12).

### 2.6 Covariate measures

Additional measures were selected based on previous research linking the covariate with neurocognitive outcomes. These included family socioeconomic status and maternal education at birth, given consistent evidence showing that these factors are correlated with child executive functioning ability (Linsell et al., 2015; Stalnacke et al., 2019; Rhoades et al., 2011; Luu et al., 2011; Hackman et al., 2015; Murtha et al., 2023). Several other family social background factors were also explored, including maternal age and marital status. However, these were not included as covariates because they did not correlate significantly with GA group status.

Family socioeconomic status (SES) was assessed using the Elley-Irving index when participants were corrected age 2 years (Elley and Irving, 2003). This measure classified families based on the highest parental occupation, into three groups: codes 1–2 = professional/managerial roles, 3–4 = technical/skilled work, 5–6 = semi- and unskilled work and unemployed. Low-family SES was defined as semi-skilled, unskilled roles and unemployed. Parental education was also recorded based on each parent’s highest qualification, ranging from 1 (did not finish high school) to 5 (university degree).

In addition, processing speed was also considered a potential covariate given findings showing that it influences how efficiently an individual can complete speeded executive tasks (Anderson, 2002; Mulder et al., 2011; Rose et al., 2011). This was measured using the Symbol Digit Modalities Test (Smith, 1973) at age 17 years. Participants used a reference key to match as many numbers with geometric figures as they could in 90 s, with the total score reflecting the correct number of substitutions made.

### 2.7 Statistical analyses

Data analysis was conducted in four steps using SPSS version 29: (1) examine the unadjusted EF scores for EPT, VPT and FT adolescents, (2) examine the impact of cognitive load on EF performance for each group, (3) examine whether expected group differences in overall EF persisted after adjusting for family social background and processing speed, and (4) examine the extent to which neonatal medical complexity and white matter abnormalities might mediate the relationship between GA and EF performance.

First, between-group differences in EF performance were examined using ANOVA, and rates of impairment were compared using chi-square tests of independence. Second, mixed ANOVAs were run with GA group as the between-subjects factor, and task level as the within-subjects factor, to examine how the different groups performed at different demand levels of each task. Third, principal components analysis and confirmatory factor analysis were conducted to assess the suitability of a single-factor model of EF for further analysis. This overall EF factor was subject to ANOVA and chi-square tests of independence as per step one, as well as ANCOVAs to control for SES, maternal education, and processing speed, and a two-way ANOVA to explore a sex by GA interaction. Finally, path analysis was conducted using the Hayes PROCESS macro to examine the extent to which associations between continuous measures of GA and overall EF performance at age 17 years might be mediated by infant neonatal medical risk over the NICU stay and/or cerebral white matter abnormalities on term MRI.

## 3 Results

### 3.1 Characteristics of the sample

Executive functioning task data were available for 36 EPT participants (missing data due to blindness, *n* = 1; severe neurodevelopmental impairment, *n* = 2, time constraints, *n* = 1, excluded due to severely impaired performance, *n* = 1), 51 VPT participants (missing data due to Cortical Visual Impairment, *n* = 1, time constraints *n* = 3), and 68 FT participants. The infant clinical and family background characteristics of the sample are presented in Table 2, with infant medical risk exposures for the EPT and VPT groups described in more detail in Supplementary Table S1. The table shows that there were expected differences in the infant clinical characteristics between the three groups (GA, birth weight, growth restriction, and plurality), but similar proportions of participants born male. The EPT group had a significantly higher neonatal medical complexity score than the VPT group (*p* < 0.001). While the EPT group had higher rates of mild to moderate white matter abnormalities than the VPT group, this trend was not statistically significant. The three groups had similar proportions of mothers who identified as New Zealand/other European, mothers who were under 21 years of age at their child’s birth, single mothers, and fathers who did not complete high school. Adolescents in the EPT and VPT groups were more likely to have been born into low SES families and to mothers who had not completed high school than FT adolescents (*p* = 0.02). EPT and VPT adolescents also had lower mean IQ and processing speed scores than their FT peers at 17 years (*p* < 0.001).

**Table 2:** Sample characteristics.

	Gestational age group				
Measure	EPT *N* = 36	VPT *N* = 51	FT *N* = 68	F/χ^2^	*p*
Infant Clinical Characteristics					
M (SD) Gestational age, weeks	26.06 (1.35)	29.76 (1.17)	39.50 (1.28)	1612.58	<0.001
M (SD) Birth weight, grams	795 (233)	1,237 (235)	3,522 (428)	1060.97	<0.001
% IUGR	19.4	5.9	1.5	11.70	0.003
% Male	58.3	47.1	50.0	1.12	0.57
% Twin	27.8	41.2	5.9	21.49	<001
M (SD) neonatal medical complexity score^a^	3.7 (2.5)	0.9 (1.6)	–	41.23	<0.001
White matter abnormality: % none	11.4	31.4	–	5.59	0.13
% mild	71.4	54.9	–		
% moderate	17.1	11.8	–		
% severe	0.0	2.0	–		
Family background characteristics					
% Mother NZ/Other European ethnicity	80.6	90.2	88.2	1.89	0.39
M (SD) Maternal age	30.97 (5.76)	30.47 (4.70)	31.37 (4.14)	11.73	0.59
% Young mother <21 years	5.6	2.0	1.5	1.68	0.43
% Single mother	22.2	15.7	10.3	2.69	0.26
% Low family SES	27.8	29.4	10.3	7.96	0.02
% Mother left school <16 years	33.3	39.2	16.2	8.45	0.02
% Father left school <16 years	34.3	32.7	23.9	1.64	0.44
17-year characteristics					
M (SD) Full Scale IQ	103.7 (9.6)	105.3 (13.8)	114 (10.9)	12.48	<0.001
M (SD) Processing speed score	45.2 (9.5)	49.4 (11.8)	53.7 (10.1)	7.78	<0.001

a A summative index including presence and severity of need for respiratory support, time taken to reach full enteral feeding, retinopathy of prematurity, neonatal sepsis, intraventricular haemorrhage/periventricular leukomalacia (IVH/PVL) and any major surgery.

### 3.2 Between-group differences in executive functioning task performance at age 17

The performance of EPT, VPT, and FT adolescents across the EF tasks is described in Table 3. As shown, there were significant linear effects of GA group on all key EF variables, with the exception of overall SOC planning time. *Post hoc* analyses showed that there were significant sub-group differences, with the EPT group performing consistently below the FT group with moderate to large effect sizes (Cohen’s *d* range: 0.58–1.03). The results also showed that the VPT group was characterized by impaired EF task performance relative to the FT group across most measures, with effect sizes predominantly in the moderate range (Cohen’s *d* range: 0.51–0.75). Although adolescents in the EPT group consistently performed less well than adolescents in the VPT group across all EF outcome measures, these between-group differences did not reach statistical significance, with the exception of the SWM task (Cohen’s *d* = 0.50 for the total errors score).

**Table 3:** Executive function performance for extremely preterm, very preterm and full term born adolescents at age 17 Years.

		Gestational age group			ANOVA *F*	*Post hoc* tests	
EF measure		I: EPT *N* = 36	II: VPT *N* = 51	III: FT *N* = 68		Sub-group differences	Cohens *d* (95% CI)
Working memory							
SSP	Forwards span	6.47 (1.30)	6.76 (1.51)	7.51 (1.20)	15.38***^a^	I & III*** II & III**	0.84 (0.42–1.26) 0.56 (0.19–0.93)
	Backwards span	5.72 (1.21)	5.92 (1.32)	6.63 (1.34)	13.33***^a^	I & III** II & III*	0.70 (0.29–1.12) 0.53 (0.16–0.90)
SWM	Total revisit errors	27.42 (17.94)	18.88 (16.76)	17.87 (15.62)	6.78**^a^	I & II* I & III*	0.50 (0.06–0.93) 0.58 (0.17–0.99)
	4-boxes	0.64 (1.50)	0.39 (1.15)	0.62 (2.20)	0.30	–	–
	6-boxes	7.72 (7.71)	4.29 (5.36)	3.99 (5.28)	5.11**	I & II** I & III***	0.53 (0.10–0.97) 0.60 (0.19–1.01)
	8-boxes	19.06 (12.20)	14.20 (12.00)	13.26 (11.38)	2.99*	I & III*	0.50 (0.09–0.91)
Planning							
SOC	Percent of perfect solutions	68.98 (17.44)	73.86 (15.99)	81.50 (14.17)	16.48***^a^	I & III*** II & III*	0.81 (0.39–1.23) 0.51 (0.14–0.88)
	2-moves	97.22 (11.61)	100.00 (00.00)	98.53 (8.51)	1.32	–	–
	3-moves	75.00 (18.36)	92.16 (18.36)	96.32 (13.15)	12.74***	I & II*** I &III***	0.68 (2.34–1.12) 0.97 (0.55–1.40)
	4-moves	66.67 (23.15)	62.25 (25.68)	73.90 (23.03)	3.56*	II & III*	0.48 (0.11–0.85)
	5-moves	54.17 (27.71)	63.24 (27.09)	73.16 (24.93)	6.41**	I & III*** II & III*	0.73 (0.32–1.15) 0.38 (0.02–0.08)
	Planning time	5443.56 (3635.87)	6318.74 (3907.61)	6518.91 (4139.95)	1.57^a^	–	–
	2-moves	2069.68 (1517.51)	2235.15 (1271.17)	2130.41 (2455.49)	0.08	–	–
	3-moves	5335.81 (3315.48)	4946.68 (3306.97)	4564.05 (3568.41)	0.54	–	–
	4-moves	7862.17 (7544.23)	8039.77 (5448.06)	8927.75 (6198.11)	0.45	–	–
	5-moves	6506.60 (5044.31)	10053.34 (10019.69)	10453.43 (7738.15)	3.05*	I & III*	0.57 (0.16–0.92)
Cognitive flexibility							
IED	Total adjusted errors^b^	23.25 (16.50)	25.06 (18.68)	18.50 (15.56)	4.18*^a^	–	–
	Intra-dimensional shift errors	7.17 (5.50)	6.10 (2.19)	6.00 (2.05)	0.41	–	–
	Extra-dimensional shift errors	10.56 (9.32)	11.36 (9.54)	8.51 (9.08)	2.72	–	–
CTMT	Composite Index T score	35.65 (9.79)	37.96 (11.10)	47.00 (11.80)	28.14***^a^	I & III*** II & III***	1.03 (0.60–1.45) 0.75 (0.37 1.13)
	Trail 1, ms	41.06 (12.64)	40.60 (16.28)	33.22 (12.96)	5.44**	I & III* II & III*	0.61 (0.19–1.02) 0.51 (0.13–0.89)
	Trail 2, ms	44.08 (15.15)	43.92 (21.56)	34.27 (11.93)	6.63**	I & III* II & III**	0.75 (0.33–1.16) 0.58 (0.20 - 0.96)
	Trail 3, ms	50.39 (13.68)	50.13 (18.72)	38.54 (13.56)	10.74***	I & III*** II & III***	0.87 (0.45–0.29) 0.73 (0.34–1.11)
	Trail 4, ms	37.44 (12.31)	36.94 (15.23)	29.22 (10.10)	7.44**	I & III** II & III**	0.75 (0.33–1.17) 0.61 (0.23–0.99)
	Trail 5, ms	67.03 (23.88)	61.44 (22.66)	48.31 (18.31)	10.76***	I & III*** II & III**	0.92 (0.419–1.34) 0.65 (0.27–1.03)
Executive function composite score		7.76 (2.02)	8.51 (2.30)	9.99 (2.00)	29.27***^a^	I & III*** II & III***	1.11 (0.68–1.54) 0.70 (0.32–1.07)

All data presented are Mean (SD). SSP, Spatial Span; SWM, Spatial Working Memory; SOC, Stockings of Cambridge; IED, Intra-Extra Dimensional Set Shift; CTMT, Comprehensive Trail-Making Test. * ≤ 0.05, ** ≤ 0.01, *** < 0.001.

a ANOVA test of linearity used.

b Results analysed using log transformed variable.

Table 4 describes the rates of EF impairment for each study group, based on the lowest 10% of the comparison group score distribution for the key variables from each EF domain. As shown, EPT-born adolescents were at greatest risk for EF impairment across all domains compared to the FT group, with relative risks ranging from 1.4 to 3.7. The VPT-born adolescent group was also at increased risk for EF impairment compared to the FT group, with relative risks ranging from 1.2 to 3.1 across domains.

**Table 4:** Rates of impairment on executive function measures for EPT, VPT, and FT born adolescents at age 17 Years.

EF measure	EPT *N* = 36	VPT *N* = 51	FT *N* = 68	*Χ^2^*	*p*
Working memory					
SSP Forwards	22.2	23.5	8.8	5.51	0.06
SSP Backwards	47.2	41.2	22.1	8.27	0.02
SWM total revisit errors	16.7	13.7	11.8	0.49	0.79
Planning					
SOC percent of perfect solutions	33.3	17.6	8.8	9.83	0.007
Cognitive flexibility					
IED total adjusted errors	22.2	25.5	11.8	3.99	0.14
CTMT composite index	38.9	37.5	11.9	13.11	0.001
Executive function composite score	50.0	29.4	10.3	19.90	<0.001

All data are presented as %. SSP, Spatial Span; SWM, Spatial Working Memory; SOC, Stockings of Cambridge; IED, Intra-Extra Dimensional Set Shift; CTMT, Comprehensive Trail-Making Test.

### 3.3 Impact of increasing cognitive demand on EF task performance

**Figure 2:**
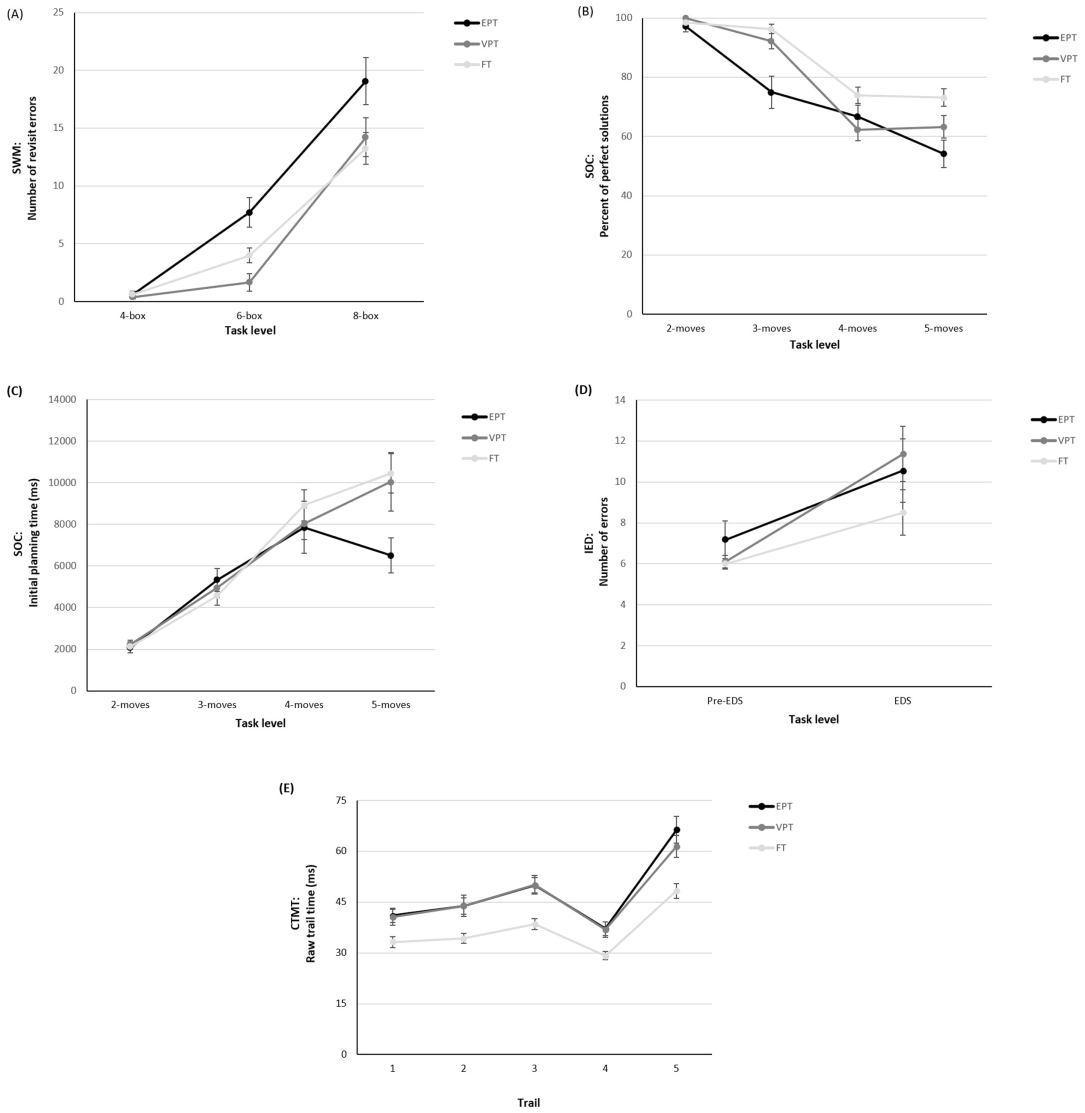
EF task performance by gestational age group and cognitive load. **(A)** SWM total revisit errors; **(B)** SOC percent of perfect solutions; **(C)** SOC initial planning time; **(D)** IED total adjusted number of errors; and **(E)** CTMT raw trail completion time across task levels.

We next examined the impact of increasing cognitive demand on EF performance for those tasks with trials of varying levels of difficulty (SWM, SOC, IED, CTMT). On the SWM task (Figure 2A), an interaction was observed between GA group and demand level that approached significance, *F*(2.77, 210.83) *=* 2.43, *p* = 0.071, partial η^2^ = 0.031, *ε* = 0.694. Follow-up analyses revealed that the EPT group made significantly more errors than the VPT and FT groups on the 6-box trials and significantly more errors than the FT group on the 8-box trials (see Table 3).

On the SOC task, there was a significant interaction between GA group and demand level for the percentage of trials solved in the minimum number of moves (Figure 2B), *F*(5.41, 411.34) = 4.70, *p* = 0.001, η^2^ = 0.058, ε = 0.902. Follow-up analyses revealed a clear between-group difference across the 3-, 4-, and 5-move trials, with both the EPT and VPT groups solving significantly fewer problems than the FT group (see Table 3). An interaction was also found for GA group and demand level on initial planning time (Figure 2C), *F*(4.26, 323.59) = 2.90, *p* = 0.02, η^2^ = 0.037, ε = 0.710, with follow-up analyses revealing that, compared to the FT group, the EPT group spent significantly less time planning on trials at the highest difficulty level (5 moves; see Table 3).

On the IED task, there was no interaction between GA group and task demand level (Figure 2D), *F*(2, 152) = 0.10, *p* = 0.91, η^2^ = 0.001. Finally, on the CTMT task, there was a significant interaction between GA group and CTMT trail on completion time (Figure 2E), *F*(5.89, 436.25) *=* 2.75, *p* = 0.01, partial η^2^ = 0.036, ε = 0.737. Follow-up analyses showed that EPT and VPT adolescents were significantly slower to complete each of the trails than the FT group (see Table 3).

### 3.4 Overall EF outcome

Given the generally consistent pattern of results across EF outcome measures, we assessed the possibility of creating a composite measure of adolescent EF to allow an examination of the extent to which later EF performance might be predicted by neonatal factors. This approach was chosen for several reasons, including that (1) empirical research with this cohort supported an underlying common EF factor at earlier ages, (2) generally consistent between-group differences were evident across all EF domains, and (3) the modest sample size precluded the inclusion of multiple latent factors. As noted in the Methods, Principal Components Analysis and Confirmatory Factor Analysis further supported the use of a single EF factor (see Supplementary material 2 for a summary of these results). Thus, a composite measure of EF was computed by summing z-scores (based on the comparison group results) from each of the key task variables. These key variables were (1) SSP forwards span, (2) SSP backwards span, (3) SWM total revisit errors, (4) SOC percent of perfect solutions, (5) IED total adjusted errors, and (6) CTMT composite index T score. Summed z-scores were then standardized (*M* = 10, SD = 2).

Before examining the role of neonatal factors in overall EF performance, we examined group differences on this overall composite, adjusting for additional covariates and known risk factors. As shown in Table 3, adolescents born EPT and VPT had significantly lower EF composite scores than the FT group, and the overall between-group difference remained following adjustment for family SES, maternal education, and processing speed (*p* = 0.002, partial η^2^ = 0.081). We also examined the role of sex at birth and found no sex by GA group interaction for this EF composite score, *F* (2, 149) = 1.56, *p* = 0.213, partial η^2^ = 0.021. Scores also did not differ by participant sex, *F* (1, 149) = 1.04, *p* = 0.310, partial η^2^ = 0.007. As shown in Table 4, EPT-born adolescents had relative risks of later overall EF impairment that were 4.9 times, and VPT-born adolescents 2.9 times, higher than FT adolescents.

### 3.5 The role of neonatal medical complexity and neonatal white matter abnormalities in executive function outcome

An unadjusted linear regression analysis showed that GA significantly predicted overall EF performance at age 17 years within the EPT and VPT groups (*β* = 0.25. *p = _._*02). To examine the role of neonatal medical complexity and white matter abnormality in potentially explaining the relationship between continuous GA and overall EF, we ran a multiple mediation model. These results are summarized in Table 5. Findings revealed a significant indirect effect of GA on EF performance through medical complexity (*b* = 0.169, *t* = −1.73) and a significant indirect effect of GA on EF performance through neonatal white matter abnormality (*b* = 0.107, *t* = −3.33). Furthermore, the direct effect of GA on EF performance in the presence of these mediators was no longer significant (*b* = −0.05, *t* = 0.38), suggesting full mediation. An examination of the individual risks included in the medical complexity score showed that the indirect relationship between GA and EF through medical complexity was driven mainly by the degree of neonatal sepsis, and including sepsis in the model in place of medical complexity revealed similar results.

**Table 5:** Mediation analysis summary.

Total effect (GA - > EF performance)	Direct effect (GA - > EF performance)	Relationship	Indirect effect	Confidence interval		*t*	Conclusion
				Lower	Upper		
0.223 (*p* = 0.04)	−0.054 (*p* = 0.70)	GA → medical complexity score → EF performance	0.169	0.152	0.370	−1.73	Full mediation
		GA → white matter class → EF performance	0.107	0.025	0.219	−3.33	Full mediation

## 4 Discussion

In this regionally representative sample of adolescents born VPT, we found that adolescents born very and extremely preterm are at increased risk of experiencing EF challenges that span working memory, planning, and cognitive flexibility domains. Consistent with existing research, the degree of prematurity significantly impacted 17-year EF performance (Lundequist et al., 2015; Taylor et al., 2004). Specifically, the EPT group performed most poorly, obtaining scores that were 0.6 to 1.0 SDs below the FT group across the individual EF measures, and 1.1 SDs below the FT group on the EF composite measure. As a result, EF impairment was relatively common, with 50% meeting criteria on our composite measure. Nonetheless, adolescents in the VPT group also showed compromised EF performance, with scores 0.5 to 0.75 SDs below the FT group across individual EF measures and the EF composite, and 29% demonstrating overall EF impairment. Between-group differences in overall EF remained unchanged following adjustment for potential confounders including family SES and maternal education.

Despite seemingly pervasive EF difficulties, between-group differences varied in magnitude across measures. Specifically, there were no differences between the VPT and FT groups on the SWM task, in contrast to previous studies of younger adolescents (Curtis et al., 2002; Fitzpatrick et al., 2016; Litt et al., 2012). However, the VPT group performed worse than the FT group on the SSP, another working memory task. This finding highlights the importance of employing multiple indicator measures for each construct of interest to avoid measurement-specific findings that might underestimate the cognitive challenges of those born VPT. This could be addressed by including multiple tasks that tap the same construct or utilizing tasks with even greater progressive difficulty.

Using the latter approach, we found that in general, VPT and EPT adolescents were characterized by progressively deteriorating performance on EF tasks with increasing cognitive demand, extending previous research conducted in childhood and early adolescence (Jaekel et al., 2013; Wehrle et al., 2016; Woodward et al., 2022). This effect was most marked for the EPT group, whose performance deviated significantly from the FT comparison group when task demands were highest. This is likely to be reflected in widening discrepancies in academic achievement in response to the increasing EF demands of secondary school (Zelazo and Carlson, 2020). Alongside school success, autonomy is central to late adolescence, and several salient and complex facets of adolescent life, such as navigating peer social relationships, gaining employment, and learning to drive, require high cognitive resources. Therefore, executive deficits that impact planning, problem-solving, and flexible thinking will present significant challenges during this developmental transition (Ben-Asher et al., 2023; Ghawami et al., 2022; Holmes et al., 2016; Pope et al., 2016).

Regarding early life predictors of the late adolescent EF abilities of those born very preterm, neonatal medical complexity and neonatal white matter abnormality class mediated the association between GA and overall EF performance at age 17 years. Concerning medical complexity, we found that a lower GA was associated with a higher medical complexity score, which, in turn, was associated with a lower overall EF score at 17 years. A similar approach to examining the additive impacts of neonatal risk factors was taken by Curtis et al. (2002), who found that a higher cumulative risk score predicted poorer spatial working memory in early adolescence. Stalnacke et al. (2019) also found an indirect relationship between cumulative risk and cognitive flexibility at 18 years.

In a secondary exploratory analysis, we found that the only individual risk factor significantly associated with later EF performance was the degree of neonatal infection. Sepsis has been shown to be an independent risk factor associated with neurodevelopmental outcomes in infancy and toddlerhood (Fibbiani et al., 2019; Rallis et al., 2019) and to middle childhood (Rand et al., 2016), yet its longer-term cognitive effects have not yet been examined. Other studies have linked individual medical factors such as oxygen requirement (Saavalainen et al., 2007; Taylor et al., 2004), and abnormal neonatal brain EEG or ultrasound (Luu et al., 2011; Saavalainen et al., 2007) with poorer EF performance in adolescence, suggesting dramatic long-term effects of inflammation, reduced cerebral blood flow and ischaemic injury on the developing brain.

In our study, sepsis was also strongly associated with the presence of the other medical risks included in the composite, meaning those individuals with sepsis also had high medical complexity scores. Further, there were relatively high rates of sepsis in the cohort compared to some of the other independent risk factors, allowing for greater statistical power to predict our EF outcome. With smaller cohorts, the ability to detect the effects of individual risk factors on later cognitive outcomes is limited, so comparing the impacts of sepsis and related individual neonatal outcomes on longer-term EF development requires further exploration.

White matter abnormalities at term equivalent also mediated the relationship between GA and 17-year EF performance. Specifically, a lower GA predicted more severe white matter pathology. In turn, more severe white matter abnormalities predicted poorer overall EF performance. Previously, white matter abnormalities were shown to predict global EF impairment at age 4 years (Woodward et al., 2011) and poorer performance across various neurocognitive domains at ages 4 and 6 years in the current cohort (Woodward et al., 2012). Similarly, others have reported an association between white matter abnormalities and cognitive ability up to age 9 years (Anderson et al., 2017; Iwata et al., 2012). There is growing evidence to suggest that early cerebral abnormalities have both primary and secondary longer-term impacts on brain development. Early diffuse white matter injury and structural changes impact subsequent gray and white matter development (Back, 2017; Boardman et al., 2006; Hüppi et al., 2001; Thompson et al., 2022), including altering functional connectivity networks, which are important for higher-order cognitive functions (Cheong et al., 2009; He and Parikh, 2015). Several studies have also shown that persisting white matter alterations present in adolescents and young adults born VPT, such as reduced fractional anisotropy in several white matter tracts and reduced white matter volume, are associated with poorer cognitive performance (Narberhaus et al., 2008; Nosarti et al., 2008; Skranes et al., 2007; Vollmer et al., 2017; Thompson et al., 2022). The current study further supports the neonatal importance of early white matter abnormalities for later neurocognitive risk, showing that the impacts extend well into adolescence.

This study had numerous strengths, including its prospective longitudinal design and a representative cohort of individuals born VPT with high retention over 17 years. In addition, we included a comprehensive battery of tasks assessing our key EF constructs of interest. Despite these strengths, several limitations are worth noting. First, we experienced quite high attrition (45%) at age 17 years in the FT comparison group. This was predominantly due to cohort members living outside the region and funding constraints. Despite this, systematic bias was unlikely given that assessed and not assessed FT study participants did not differ on social background measures at age 17.

Second, despite good retention and representativeness in the very preterm group, we were limited in the number of variables included in the statistical analysis because of the modest sample size. A key focus of this paper was the neonatal predictors of later EF risk. Given that these neonatal factors were found to fully mediate the effects of GA at birth on later EF, other postnatal factors, such as parenting and family functioning factors, were not included in the mediation model. We also did not include postnatal brain MRI measures since the model was fully mediated by neonatal factors. However, future research should examine how aspects of parenting and/or early or school-based intervention might help improve EF skills following VPT birth in this older age group.

In conclusion, findings indicate that adolescents born VPT and EPT were more likely to experience EF difficulties across working memory, planning and cognitive flexibility domains, especially when task demands were high. Associations between GA and EF outcome were fully mediated by neonatal medical complexity (predominantly neonatal infection) during the NICU stay and the presence/severity of cerebral white matter abnormalities at term equivalent. This supports the importance of post-discharge monitoring and early intervention at least to school age for very preterm infants subject to a complex medical course, so as to ensure likely longer-term challenges with EF can be detected and addressed to optimize longer-term outcomes. In addition, screening for white matter abnormalities at term may assist with identifying individuals born very preterm who are at risk of persistent neurocognitive difficulties and aid, or at least help justify, longer-term surveillance and support.

## Data Availability

The raw data supporting the conclusions of this article will be made available by the authors, without undue reservation.
