# Supplementary material for: Executive functioning challenges of adolescents born extremely and very preterm
Source: Front Psychol. 2024 Dec 11;15:1487908. doi: 10.3389/fpsyg.2024.1487908 (PMC11669177; doi:10.3389/fpsyg.2024.1487908)
Supplement: Supplementary file 1 [file Table_1.DOCX]

Supplementary Material

# Infant Medical Exposures for EPT and VPT participants

| Table S1. Infant Medical Exposures for EPT and VPT Participants | | | | | |
| --- | --- | --- | --- | --- | --- |
| **Exposure** | **Severity (Score)** | **EPT**  **N=36**  **%** | **VPT**  **N=51**  **%** | ***t/*χ^2^** | ***p*** |
| Respiratory support | 28 days – 36 weeks (1) | 25.0 | 7.8 | 40.37 | <.001 |
|  | O^2^ at 36 weeks or dex (2) | 61.1 | 9.8 |  |  |
| Full enteral feeds | 11-21 days (1) | 36.1 | 9.8 | 18.78 | <.001 |
|  | >21 days (2) | 13.9 | 0.0 |  |  |
| ROP | Stage 3 with Rx (2) | 5.6 | 0.0 | 2.9 | .09 |
| Sepsis | Multiple BC– (1) | 16.7 | 3.9 | 17.30 | <.001 |
|  | BC+ or NEC (2) | 41.7 | 11.8 |  |  |
| IVH | Grade 3 resolved PVL flare (1) | 11.1 | 2.0 | 3.30 | .19 |
|  | Grade 3 with HC, grade 4, or PVL (2) | 2.8 | 3.9 |  |  |
| Surgery | Any surgery (2) | 19.4 | 5.9 | 3.82 | .05 |
| Complexity group: | 1 (total score 0-2) | 22.2 | 74.5 |  |  |
|  | 2 (total score 3-4) | 44.4 | 21.6 |  |  |
|  | 3 (total score 5+) | 33.3 | 3.9 | 25.82 | <.001 |
| Note: ROP, retinopathy of prematurity; Rx, prescription; BC, blood culture; NEC, nectrotizing enterocolitis; IVH, intraventricular haemorrhage; PVL, periventricular leukomalacia; HC, hydrocephalus | | | | | |

# Results: Principle Components Analysis and Confirmatory Factor Analysis

A principal components factor analysis (PCA) was used to assess the factor structure underlying the six observed EF measures (SSP forwards, SSP backwards, SWM total errors, SOC perfect solutions, IED total adjusted errors, and CTMT composite index T score). Inspection of the correlation matrix showed that all variables had at least one correlation coefficients greater than 0.3.The Kaiser-Meyer-Olkin value was 0.83, exceeding the recommended value of 0.6. Bartlett’s Test of Sphericity reached statistical significance (*p*<.001), indicating that the data were likely factorizable. PCA supported the presence of a single EF component with an eigenvalue exceeding 1 and explaining 49% of the total variance. Visual inspection of the scree plot also showed a clear break after the first component. Therefore, one EF component was retained for further investigation. Results were similar when using the entire sample, the EPT/VPT group only, and the FT group only.

Confirmatory factor analysis (CFA) was then performed using the entire sample to confirm the viability of a single factor model of EF. AMOS Graphics was used to create a path analysis diagram to examine the model fit of the EF measures, with EF as the latent variable, and the EF tasks as the observed variables. The regression weights show the correlations between the latent and observed variables ranging from -.41 to .79. The remaining factor loadings reflect how much of the observed variable is explained by the latent variable ranging from .17 to .62. The model had good fit (χ^2^_9_ = 12.67, *p* = .18; comparative fit index (CFI) = .99; root mean-square error of approximation (RMSEA) = .04 [90% confidence interval = .00–.09, *p-­*value RMSEA = .54]).
